# Radiation-induced angiosarcoma of the breast: a retrospective analysis of 15 years' experience at an oncology center

**DOI:** 10.1590/0100-3984.2017.0129

**Published:** 2018

**Authors:** Ines Alves, José Carlos Marques

**Affiliations:** a Department of Radiology, Hospital Central do Funchal, Funchal, Portugal.; b Department of Radiology, Instituto Português de Oncologia de Lisboa Francisco Gentil, Lisboa, Portugal.

**Keywords:** Breast neoplasms, Hemangiosarcoma/diagnosis, Neoplasms, radiation-induced, Radiotherapy, Tertiary care centers, Neoplasias da mama, Hemangiossarcoma/diagnóstico, Neoplasias induzidas por radiação, Radioterapia, Centros de atenção terciária

## Abstract

**Objective:**

To characterize a population of patients with radiation-induced angiosarcoma (RIAS) of the breast treated at an oncology center, focusing mainly on the imaging features, although also on the clinical presentation, diagnosis, and management.

**Materials and Methods:**

We performed a retrospective review of patients with histologically proven angiosarcoma of the breast or chest wall, all of whom received radiotherapy, after conservative or radical breast surgery, between 2000 and 2015.

**Results:**

Eleven patients met the inclusion criteria. The median age at the time of diagnosis of RIAS of the breast was 71.5 years (range, 58-87 years), and the median latency period was 8.9 years (range, 4-27 years). The rate of local recurrence was 54.4%, RIAS recurring after a median period of 10 months (range, 3-18 months), and distant metastases occurred in three patients (27.3%). All of the tumors were accompanied by skin changes, and a palpable mass was seen in four. Most of the imaging findings were nonspecific. Six patients underwent magnetic resonance imaging, which revealed pronounced skin enhancement in all six. Ultrasound-guided core needle biopsies were negative in three of the eight patients.

**Conclusion:**

RIAS of the breast is a rare but recognized complication of radiotherapy for breast carcinoma, with a poor prognosis and high recurrence rate, which requires a high index of suspicion for a prompt diagnosis.

## Abstract (pt)

**Objetivo:**

Caracterizar uma população de pacientes com angiossarcoma da mama induzido por radioterapia em um instituto de oncologia, com foco principalmente nas características de imagem, mas também na apresentação clínica, diagnóstico e gerenciamento.

**Materiais e Métodos:**

Efetuamos análise retrospectiva, entre 2000 e 2015, de pacientes com angiossarcoma da mama ou parede torácica histologicamente comprovado que foram previamente irradiadas após cirurgia mamária, conservadora ou radical.

**Resultados:**

Onze pacientes preencheram os critérios de inclusão. A mediana da idade na ocasião do diagnóstico de angiossarcoma da mama induzido por radioterapia foi 71,5 anos (variação: 58-87 anos) e o período médio de latência foi 8,9 anos (variação: 4-27 anos). A taxa de recorrência local foi 54,4%, após mediana de 10 meses (variação: 3-18 meses), e metástases a distância ocorreram em três pacientes (27,3%). Todos os tumores apresentaram alterações na pele, e massa palpável observou-se em quatro. Os achados de imagens foram globalmente inespecíficos. Os seis paciente que realizaram ressonância magnética revelaram aumento de captação de contraste cutâneo. As biópsias ecoguiadas foram negativas em três de oito pacientes.

**Conclusão:**

Angiossarcoma da mama induzido por radioterapia é complicação rara, mas conhecida, da radioterapia para câncer da mama, com mau prognóstico e alta taxa de recorrência, sendo necessário elevado índice de suspeição para seu diagnóstico imediato.

## INTRODUCTION

Angiosarcoma of the breast is a malignant tumor with a vascular endothelial origin that can be either primary, if it arises without a known precursor, or secondary, if it occurs at the site of previously irradiated skin, in which case it is known as a radiation-induced angiosarcoma (RIAS) of the breast^(1)^. Although RIAS of the breast is rare, accounting for < 1% of breast tumors^(2)^, it is a documented complication of radiotherapy for breast cancer, with a high recurrence rate and poor outcomes. It accounts for approximately about 3% of all soft-tissue sarcomas^(3)^.

The diagnosis of RIAS of the breast is often delayed because of its benign appearance and the difficulty in differentiating it from the nonspecific skin changes induced by prior radiation. The radiologic findings are also often nonspecific. Because the incidence of breast cancer is increasing, the use of breast-conserving surgery followed by radiotherapy has been replacing radical mastectomy as the standard treatment. The associated incidence of RIAS is also rising, with an estimated cumulative incidence of 0.9-3.2 per 1000 breast cancer cases over 15 years^(3,4)^.

Although RIAS typically develops 10 years after radiation treatments for breast cancer, the latency period can range from 6 months to 20 years^(5)^, leading some authors to believe that it is probably being underreported and that the true incidence rates are therefore higher^(2)^. Despite the fact that RIAS was first described in the early 1920s, its molecular biology is still controversial and there is therefore no targeted therapy available^(5)^. Currently, aggressive surgical resection is commonly advocated as the treatment of choice. There is a lack of data proving the efficacy of adjuvant chemotherapy^(6)^.

We have performed a retrospective study to characterize a population of patients with RIAS treated at a tertiary care hospital over a 15-year period, analyzing the usefulness of mammography, ultrasound, and magnetic resonance imaging (MRI) in making the diagnosis. Clinical and pathological findings were also reviewed.

## MATERIALS AND METHODS

This was a retrospective, single-center study. The study was approved by the local institutional review board, and the requirement for informed consent was waived because of the retrospective nature of this study. The affected patients were identified through a comprehensive search of the cancer registry of our institution.

We performed a retrospective analysis of patients with histologically proven angiosarcoma of the breast or chest wall who underwent radiotherapy after conservative or radical breast surgery, between 2000 and 2015. For most of the cases, the doses and modality of the radiotherapy were unknown. All the specimens were evaluated by a pathologist specializing in breast diseases.

To diagnose RIAS, we followed the Cahan et al. criteria, as modified by Arlen et al., for radiation-associated neoplasms^(7)^: sarcoma arising within the previous irradiated field; a latency period of at least 3 years between radiotherapy and the development of the sarcoma; and a histological distinction between the secondary sarcoma and the primary neoplasm. Patients with Stewart-Treves syndrome were excluded. After the study criteria had been applied, the study sample comprised 11 female patients.

The date of RIAS diagnosis was defined as the day on which the histological diagnosis was made. The latency period was defined as the time from the final radiation session and the date of RIAS diagnosis. The tumor grade was based on the pathology report at the time of the initial RIAS diagnosis. Local recurrence was defined as the reappearance of a tumor at the initial site, without metastasis.

## RESULTS

### Patient characteristics

The cohort included 11 women with a median age of 71.5 years (range, 58-87 years) at the time of the RIAS diagnosis. The median latency period for developing a second malignant angiosarcoma was 8.9 years (range, 4-27 years). The underlying (breast cancer) diagnosis was invasive ductal carcinoma in nine patients and ductal carcinoma *in situ* in two.

All of the patients presented with alterations on physical examination: seven had only skin lesions; and four had skin lesions and a palpable mass. The most common skin alterations were discoloration (blue or red) and papules.

In most of the cases, the diagnosis was made promptly, on the basis of the characteristic skin changes and subsequent core needle biopsy results. However, in four patients the correct diagnosis took 2-6 years to achieve, because the clinical findings were uncharacteristic and consecutive biopsies were not diagnostic.

Ten of the 11 patients underwent mastectomy as the initial treatment. Out those ten patients, four had wide local excisions (three underwent removal of all or part of the pectoralis major muscle, and one underwent chest wall resection). In addition to surgical resection, five patients received chemotherapy (one for the treatment of the primary RIAS, because of locally advanced disease, and four for post-resection recurrence) and one underwent a second round of radiotherapy, as palliative treatment after RIAS recurrence.

Recurrence disease was found in 6 (54.4%) of the 11 patients experienced recurrence, after a median of 10 months (range, 3-18 months). Distant metastases occurred in three patients (27.3%): at the time of RIAS diagnosis in one and subsequent to recurrence in two.

Of the 11 patients evaluated, 7 died within the first 1-6 years (mean, 3.1 years) after RIAS diagnosis. According to the death certificates, the tumor was the underlying cause of death in at least five of those cases. The 5-year survival rate was 45%. The features of the studied population and the RIAS characteristics are shown in Table 1.

**Table 1:** Features of the study population and characteristics of RIAS.

Case	Age (years)	Latency period (years)	Skin changes	Biopsy	Histological grade	Recurrence	Metastases	Fatal course
1	60	7	Skin discoloration + nodule with skin retraction	Positive	G2	Positive	Negative	Negative
2	87	7	Skin discoloration + ulcers + papules	Negative	G3	Positive	Negative	Negative
3	82	7	Huge ulcers + papules	Positive	G2	Negative	Negative	Negative
4	60	11	Papules	Negative	G3	Negative	Negative	Negative
5	73	27	Skin discoloration + huge hemorrhagic papules	Inconclusive	G3	Negative	Negative	Positive
6	72	11	Nodule with inflammatory signs	Positive	G3	Negative	Positive	Positive
7	58	9	Skin discoloration	Inconclusive	G3	Positive	Positive	Positive
8	77	4	Extensive papules	Positive	G3	Negative	Negative	Positive
9	59	6	Nodule + ulcers	Inconclusive	G3	Positive	Positive	Positive
10	81	4	Skin discoloration	Positive	G3	Positive	Negative	Positive
11	77	5	Nodule + ulcers	Negative	G2	Positive	Negative	Positive

### Radiological findings

In most cases, the radiological presentation of the RIAS was nonspecific. Skin changes and nodules were the main clues to diagnosis in the majority of cases. In three of the patients, there were no radiological findings suggestive of the diagnosis. The ultrasound-guided core needle biopsies also lead to misdiagnosis: in three of the eight patients who underwent those procedures, the biopsy results were negative for RIAS and the subsequent (postoperative) histological analyses produced positive results.

#### Mammography

**Figure 1:**
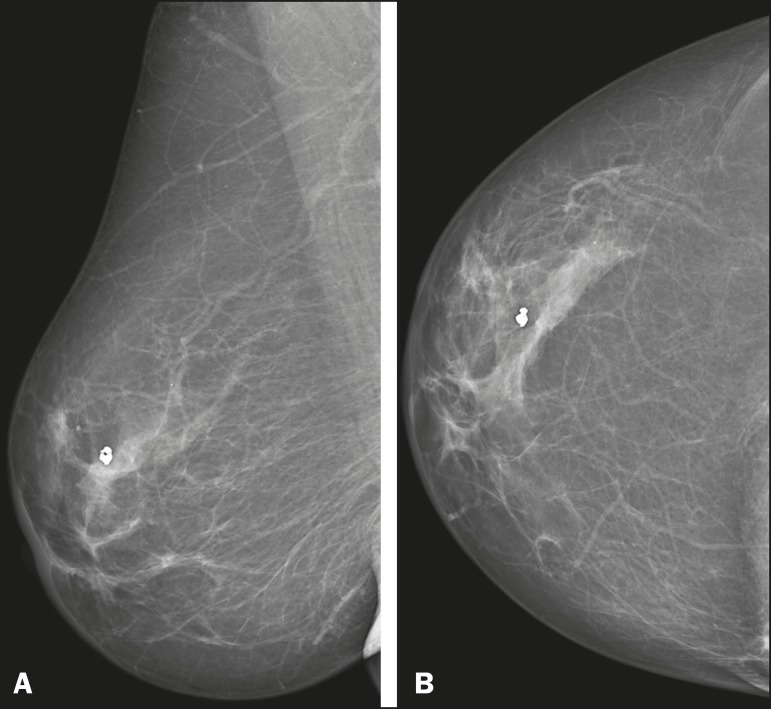
58-year-old female with high-grade RIAS. Mediolateral oblique and craniocaudal mammograms of the right breast (**A** and **B**, respectively) showing an ill-defined asymmetric lesion in the upper-outer quadrant, with skin thickening and course calcifications.

**Figure 2:**
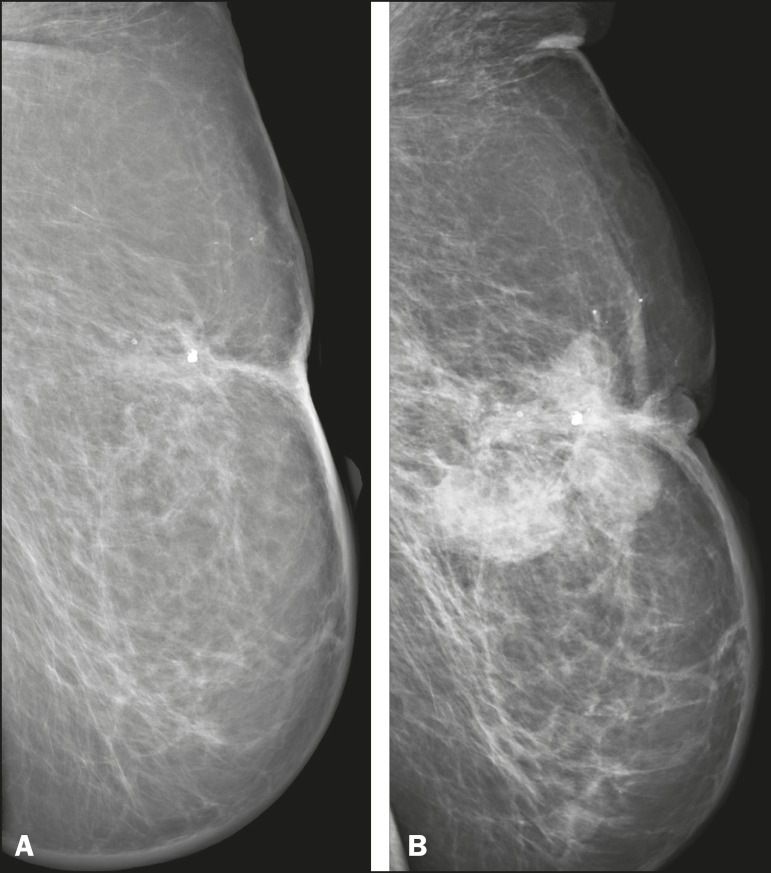
77-year-old female with intermediate-grade RIAS presenting as nodules and ulcers. Mediolateral oblique mammograms of the left breast showing hyperdense areas underlying scar tissue, accompanied by a rounded, well-circumscribed mass that was not visible in the previous examination, together with coarse calcifications and skin thickening.

Mammograms were obtained in only eight patients (four symptomatic patients and four asymptomatic patients), because the other patients had masses that were too large or painful to allow a mammogram to be performed. Of those eight patients, four were symptomatic. Two of the symptomatic patients were initially identified by detection of a mass on mammography (Figure 1). In the other two, mammograms showed suspicious findings, mainly a high-density opacity (Figure 2). In the four remaining patients (two of whom had significant skin changes), mammography depicted only nonspecific changes, such as diffuse skin thickening consistent with previous surgery.

#### Ultrasound

**Figure 3:**
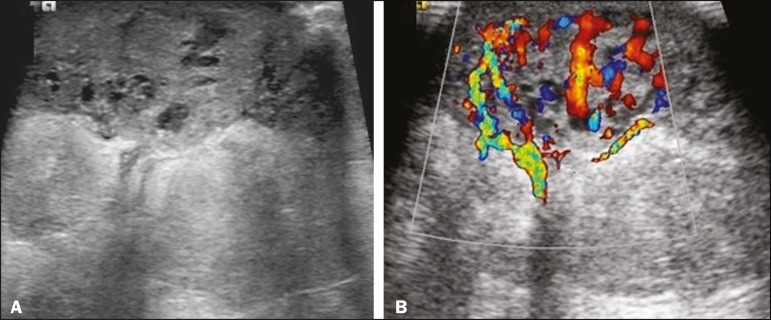
82-year-old female with high-grade RIAS and extensive papules. Gray-scale and color Doppler ultrasound of the left breast (**A** and **B**, respectively), showing a central superficial heterogeneous mass with some cystic areas and increased blood flow.

**Figure 4:**
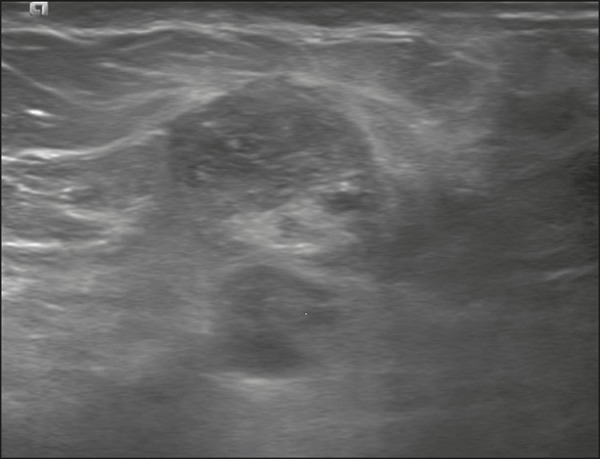
72-year-old female with high-grade RIAS, presenting with a nodule with signs of inflammation. Ultrasound of the right breast, showing multiple solid, rounded, well circumscribed masses with heterogeneous echogenicity.

Seven ultrasound scans were available for review. Four patients, all of them with accompanying mammogram findings, showed a suspicious lesion or mass, one of which was a partly exophytic mass (Figures 3 and 4). The other three patients revealed iatrogenic/nonspecific changes, including a small cystic lesion.

#### MRI

**Figure 5:**
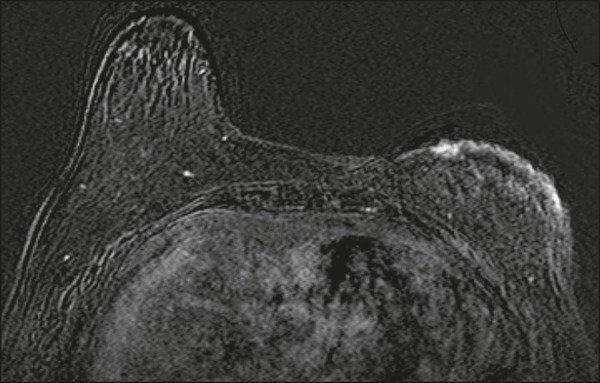
58-year-old female with high-grade RIAS who presented with mild skin discoloration. Axial sagittal gadolinium-enhanced fat-suppressed T1-weighted MRI scan showing anomalous skin enhancement on the left breast.

**Figure 6:**
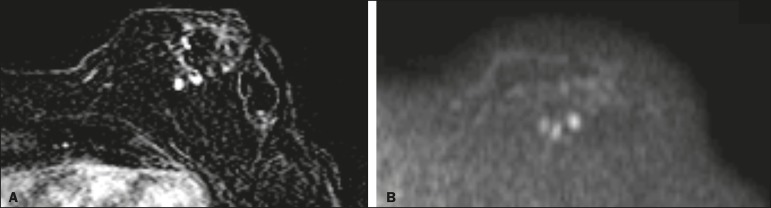
81-year-old female with high-grade RIAS who presented with extensive skin discoloration. Axial T1-weighted, gadolinium-enhanced, fat-saturated MRI sequence after subtraction (**A**) and axial diffusion-weighted (b = 1000) MRI sequence (**B**), both depicting retroareolar lesions with early, avid enhancement and restricted diffusion, highly suggestive of recurrence.

**Figure 7:**
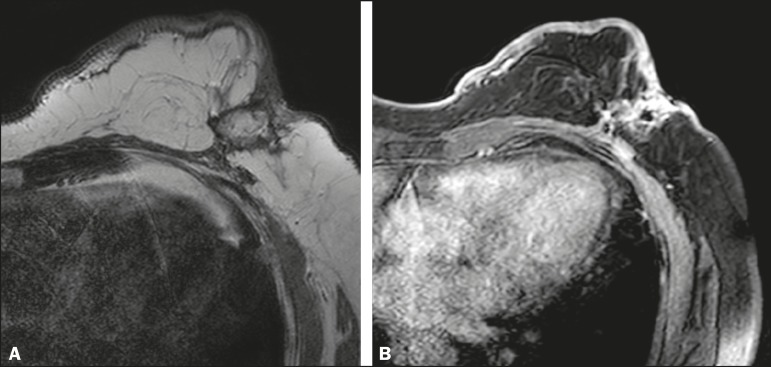
59-year-old female with high-grade RIAS who presented with a nodule and ulcers. Axial T2-weighted MRI sequence (**A**) and axial T1-weighted, gadoliniumenhanced, fat-saturated MRI sequence after subtraction (**B**), both showing enhancement of the skin and subcutaneous tissue down to the pectoralis fascia, without invasion of the muscle.

Six of the patients underwent MRI. In all six, the MRI revealed suspicious skin enhancement in addition to the expected iatrogenic findings, such as skin thickening (Figure 5). In addition, two patients showed focal enhancement of the subcutaneous tissue down to the pectoralis fascia (Figure 6): two very small lesions with rapid initial enhancement, followed by washout, in one (Figure 7); and a very large exophytic mass with heterogeneous enhancement in the other.

## DISCUSSION

In this retrospective study, we have described the clinical and imaging presentation of RIAS of the breast, as well as the diagnosis, management, and treatment outcomes of 11 patients who developed RIAS after undergoing radiotherapy for breast cancer at an oncology center between 2000 and 2015. In our patient sample, the median age at RIAS presentation was 71.5 years (range, 58-87 years), comparable to the 70 years (range, 36-92 years) reported in the literature^(8)^. The median latency period observed in our study (8.9 years) was slightly longer than the approximately 6 years reported by other authors^(4,8,9)^. That is probably due, in part, to the fact that the latency period was uncommonly long (27 years) in one of our patients.

Like other breast neoplasms, RIAS is diagnosed by physical examination combined with imaging modalities, although both can provide highly nonspecific findings^(5)^. Skin changes related to RIAS include bluish or purplish discoloration, mimicking a hematoma, as well as palpable tumors, violaceous plaques, or erythematous nodules^(6)^. Lesions can be single or multiple and vary greatly in size, ranging from small nodules to a large lesion covering the entire breast^(10)^. Such lesions usually develop on the skin/scar tissue overlying the primary cancer treatment site, overlapping with the appearance of irradiated areas^(8)^. As in other studies^(2)^, all of our patients showed abnormalities on physical examination, although, as expected, the findings were mainly nonspecific and often clinically similar to those of benign lesions.

Many of the imaging features of RIAS of the breast remain unknown, because most studies have evaluated a mix of primary and secondary breast angiosarcomas, as well as because those reviewing secondary subtypes are mostly case reports or small-scale studies that do not focus on radiological findings^(11,12)^. The imaging features of RIAS of the breast are usually nonspecific and are related to the expected iatrogenic changes; its diagnosis can therefore depend upon a high index of suspicion^(9)^. Mammography can reveal skin thickening, focal asymmetry, increased density, or a mass^(8,10)^. However, many false-negative results are reported, and even after skin changes, mammography might be negative^(5)^. Some authors have stated that approximately 33% of RIAS patient mammograms appear completely normal^(10)^. Luini et al.^(9)^ reviewed 16 cases of primary or secondary breast angiosarcomas and noted mammographic and clinical findings in all patients, most of those being nonspecific findings, such as ill-defined masses or skin thickening. Morgan et al.^(4)^ analyzed 33 RIAS patients, reporting that only two were initially diagnosed by mammography and that five symptomatic patients had unsuspicious mammographic findings.

Ultrasound does not show pathognomonic characteristics, and provides added value only in cases in which mammogram findings are also present. It can reveal a hypoechoic, hyperechoic, or heterogeneous mass, occasionally with posterior acoustic shadowing^(10)^. In our study, all depicted masses presented positive mammogram findings and nonspecific ultrasound findings.

Although there is still little information related to the use of MRI in RIAS, there have been studies demonstrating its ability to detect mammary lesions^(11)^. It also facilitates the preoperative planning of the surgical approach, determining the tumor spread, and predicting chest wall involvement^(5)^. Chikarmane et al.^(12)^, who conducted the largest cohort study of pre-treatment breast MRI of RIAS, involving patients with pathologically proven RIAS, found that 13 of the 16 patients evaluated had diffuse heterogeneous skin enhancement, with or without cutaneous, enhancing masses, and four had intraparenchymal involvement, characterized by irregular masses. After gadolinium injection, those masses revealed rapid enhancement with washout. In a case report, Vuille-dit-Bille et al.^(13)^ showed skin thickening with heterogeneous enhancement, with no intraparenchymal nodularity or mass, in a patient with RIAS. Our results underscore those of these studies, given that all of our patients had skin thickening and enhancement, with or without other findings.

Ultrasound-guided core needle biopsies results should be carefully reviewed, because they might not be diagnostic and the results can be misleading. Chen et al.^(14)^ reported a false-negative biopsy result rate of 37%, similar to the 37.5% observed in our sample. That is thought to be attributable to inadequate samples or to post-irradiation skin showing histologic features that overlap with those of RIAS^(15)^. Moe et al.^(16)^ reported the case of a RIAS patient with a short latency period and false-negative biopsy results, explaining that the borders of an angiosarcoma frequently exhibit low-grade changes that may not be distinguishable from those identified in previously irradiated tissue. Therefore, a negative biopsy can delay diagnosis and treatment, potentially resulting in a poorer prognosis^(15)^.

RIAS is known to have a high recurrence rate. Local recurrence, either in the tumor bed or along the surgical scar, is reportedly detected in a majority (96%) of cases of RIAS^(17)^. That is probably due to multifocal growth of the RIAS or remnants of malignant tissue after surgery, even with negative surgical margins^(6)^. Metastatic disease-most frequently to the lungs and the liver-can be present at the same time or shortly after local recurrences^(17)^. Abbott et al.^(8)^ retrospectively reviewed all cases of RIAS published as of 2017 and summarized the data. The authors found that the local recurrence rate was 59% and that the median time to recurrence was 6 months (range, 1-78 months). They also found that metastatic disease was usually preceded by at least one local recurrence and that the most common locations were the lungs, the contralateral breast, and the skeleton. Furthermore, their histological review of 42 cases suggested that poorly differentiated RIAS was associated with a high risk of metastasis, whereas well-differentiated tumors presented a higher risk for local recurrence rather than for distant metastases.

Despite all therapeutic efforts, the prognosis of RIAS is still poor. The reported five-year survival rate varies widely, ranging from 27% to 62.8%^(2,3,8,18,19)^. Given its aggressive behavior, extensive surgical resection, including all irradiated tissue and widely negative margins, is currently advocated. Recent studies have suggested that hyperfractionated radiotherapy can be useful in preventing recurrences^(6,10)^. However, despite the increasing number of studies focusing on chemotherapy and radiotherapy, whether neoadjuvant or adjuvant, their role remains unclear^(5)^.

This study has several limitations. First, the small patient population and the retrospective nature of the study prevented us from drawing any definitive conclusions. Studies involving larger patient samples are needed in order to confirm the results. Because ours is a tertiary care center, most of the patients had already undergone at least some imaging examinations prior to admission. However, the results of those examinations were not available in all cases. Finally, information related to the type and dose/energy of the radiation received were available for only a few patients.

## CONCLUSION

Although the incidence of RIAS followed by treatment with conservative surgery and radiotherapy is low, it has a significant impact on survival, because its overall prognosis is poor^(15)^. Because of the benign appearance of the tumor in its initial stages and the nonspecific radiologic findings, early diagnosis can be made only on when there is a high index of suspicion, careful periodic physical evaluation, and an adequate biopsy sample^(5)^. Given that local recurrence rates are high, close follow-up and investigation of even subtle skin changes of the breast are recommended for the prompt detection of recurrence^(2)^.
